# Selective JAK2 pathway inhibition enhances anti-leukemic functionality in CD19 CAR-T cells

**DOI:** 10.1007/s00262-024-03927-8

**Published:** 2025-02-01

**Authors:** Kohei Mitsuno, Masaya Suematsu, Yuki Naito, Azusa Mayumi, Hideki Yoshida, Shinya Osone, Toshihiko Imamura, Yozo Nakazawa, Shigeki Yagyu, Tomoko Iehara

**Affiliations:** 1 Department of Pediatrics, Graduate School of Medical Science, Kyoto Prefectural University of Medicine, Kyoto, Japan; 2 Department of Pediatrics, Shinshu University School of Medicine, Matsumoto, Japan; 3 Institute for Biomedical Sciences, Interdisciplinary Cluster for Cutting Edge Research, Shinshu University, Matsumoto, Japan; 4 Innovative Research and Liaison Organization, Shinshu University, Matsumoto, Japan

**Keywords:** CAR-T cell therapy, JAK2 inhibitor, Memory T cell fraction, Ph-like ALL

## Abstract

The integration of molecular targeted therapeutics with chimeric antigen receptor T (CAR-T) cell therapy represents a novel strategy to amplify the anti-tumor efficacy of immunotherapy. While CD19-targeted CAR-T cells and Janus kinase (JAK) inhibitors have independently shown efficacy against certain B-cell leukemias, such as Philadelphia chromosome-like acute lymphoblastic leukemia, the concurrent use of JAK1/2 inhibitors, such as ruxolitinib, has been implicated in reducing CAR-T cell potency by inhibiting the JAK1-dependent T cell activation pathway. This study explores the combinatorial use of a selective type II JAK2 inhibitor, CHZ868, with CD19 CAR-T cells, revealing a synergistic enhancement of anti-leukemic activity across B-cell tumor models irrespective of JAK2 mutational status. CHZ868-mediated JAK2 inhibition did not induce the exhaustion of CAR-T cells, maintaining efficacy over repeated tumor challenges and significantly extending survival in mouse models engrafted with JAK2 inhibitor-resistant leukemia cells (median survival, CD19 CAR-T + CHZ868 vs. CD19 CAR-T + DMSO: 32 days vs. 26 days, *p* = 0.0303). Transcriptomic analyses suggest that CHZ868 impedes CAR-T cell differentiation while preserving their proliferative capacity, a crucial factor in maintaining CAR-T cell functionality. Therefore, the selective inhibition of the JAK2 pathway may potentiate CAR-T cell therapy and offer a viable treatment strategy for patients with resistant B-cell leukemias.

**Supplementary Information:**

The online version contains supplementary material available at 10.1007/s00262-024-03927-8.

## Introduction

The treatment outcomes for pediatric B-cell acute lymphoblastic leukemia (B-ALL) have improved significantly in recent years. However, a subset of B-ALL cases remains treatment-resistant, necessitating a deeper understanding of their molecular biology and the development of optimized therapies. Molecular biological studies of refractory B-ALL have identified a subgroup characterized by a gene expression pattern similar to that of Philadelphia chromosome-positive (Ph +) ALL, but lacking the Ph chromosome, termed Ph-like ALL. This subtype is driven by the activation of specific signaling pathways that contribute to leukemogenesis [1]. Treatment approaches using tyrosine kinase inhibitors (TKIs) tailored to the gene expression profile of each ALL subtype have been explored [1, 2]. However, TKIs are limited by their variable efficacy depending on the genetic background of the tumor and the acquisition of resistance through genetic mutations [2–4].

Conversely, CD19 chimeric antigen receptor T cell (CAR-T) therapy, which involves T cells engineered to express a chimeric antigen receptor (CAR) that specifically binds to CD19, has shown strong and sustained anti-tumor effects against CD19-positive B-cell malignancies. Despite its efficacy, CAR-T cell therapy is associated with significant challenges, such as a high incidence of cytokine release syndrome (CRS) in patients with a high tumor burden [5, 6] and the potential for immune evasion, leading to diminished anti-tumor effects [7, 8]. Considering these challenges, combinatorial therapeutic strategies are currently under investigation. For instance, dasatinib and trametinib are known to suppress T cell activation in vitro but have demonstrated sustained anti-tumor effects in vivo by preventing T cell exhaustion caused by excessive antigen stimulation [9, 10].

Ruxolitinib, a JAK1/2 inhibitor, has been tested in Ph-like ALL which is characterized by JAK-STAT pathway activation [11]. Because ruxolitinib can suppress T cell-mediated immune responses, the combinatorial use of ruxolitinib and CD19 CAR-T cells for JAK pathway-mutated-ALL could jeopardize CAR-T cell function. Consequently, ruxolitinib has been used in severe cases of CRS to manage cytokinemia, although its use is restricted to brief durations to avoid diminishing the overall anti-tumor efficacy [12–14]. Recently, more selective JAK2 inhibitors have been developed and used for the treatment of myeloproliferative neoplasms. Given that many Ph-like ALL cases with activated JAK-STAT pathways involve JAK2 mutations, these inhibitors have a potential therapeutic value. Unlike JAK1 and JAK3, which are involved in common gamma receptor-dependent cytokine signaling crucial for T cell-mediated immunity, selective JAK2 inhibitors are hypothesized to have minimal impact on CAR-T cell activity [15]. Therefore, we aimed to investigate the therapeutic effects of CHZ868, a selective JAK2 inhibitor, in combination with CD19 + CAR-T cells against B-ALL.

## Materials and methods

### Blood sample collection and cell lines

Human peripheral blood mononuclear cells (PBMCs) were harvested from whole blood samples by density gradient centrifugation in lymphocyte separation medium 1077 (FUJIFILM Wako Pure Chemical Corporation, Osaka, Japan). After isolation, the cells were serially washed in Dulbecco’s Phosphate-Buffered Saline (D-PBS; Nacalai Tesque Inc., Kyoto, Japan). Viability and cell count were assessed by the trypan blue exclusion assay using an automated cell counter (Model R1; Olympus, Tokyo, Japan). The human B-cell precursor leukemia cell line with wild-type JAK2 (REH (RRID:CVCL_1650)) was acquired from the American Type Culture Collection (ATCC, Manassas, VA, USA). YCUB-5 (RRID:CVCL_A085), a JAK2 mutant Ph-like ALL cell line, was obtained from Kanagawa Children’s Medical Center. KOPN-49 (RRID:CVCL_W276), a JAK2 mutant Ph-like ALL cell line, was provided by the Department of Pediatrics at the University of Yamanashi. A piggyBac (PB) transposon system harboring pIRII- firefly luciferase (FFLuc)-puroR- green fluorescent protein (GFP) was introduced into the cells, followed by isolation of transduced cells using fluorescence-activated cell sorting (FACS) to generate REH cells expressing FFLuc and GFP. All cell lines were propagated in RPMI-1640 medium (Nacalai Tesque Inc.) supplemented with 10% fetal bovine serum (FBS; Thermo Fisher Scientific Inc., Waltham, MA, USA), and cultured under a humidified atmosphere of 5% CO_2_ at 37 °C.

### Pharmacologic agents

The JAK1/2 inhibitor, ruxolitinib, and the selective JAK2 inhibitor, fedratinib, were purchased from Selleckchem (Houston, TX, USA). Another selective JAK2 inhibitor, CHZ868, was purchased from Abcam (Cambridge, UK) and MedKoo (Morrisville, NC, USA). All drugs were solubilized in DMSO to prepare stock solutions, according to the manufacturer’s protocol.

### Plasmid construction

**Fig. 1:**
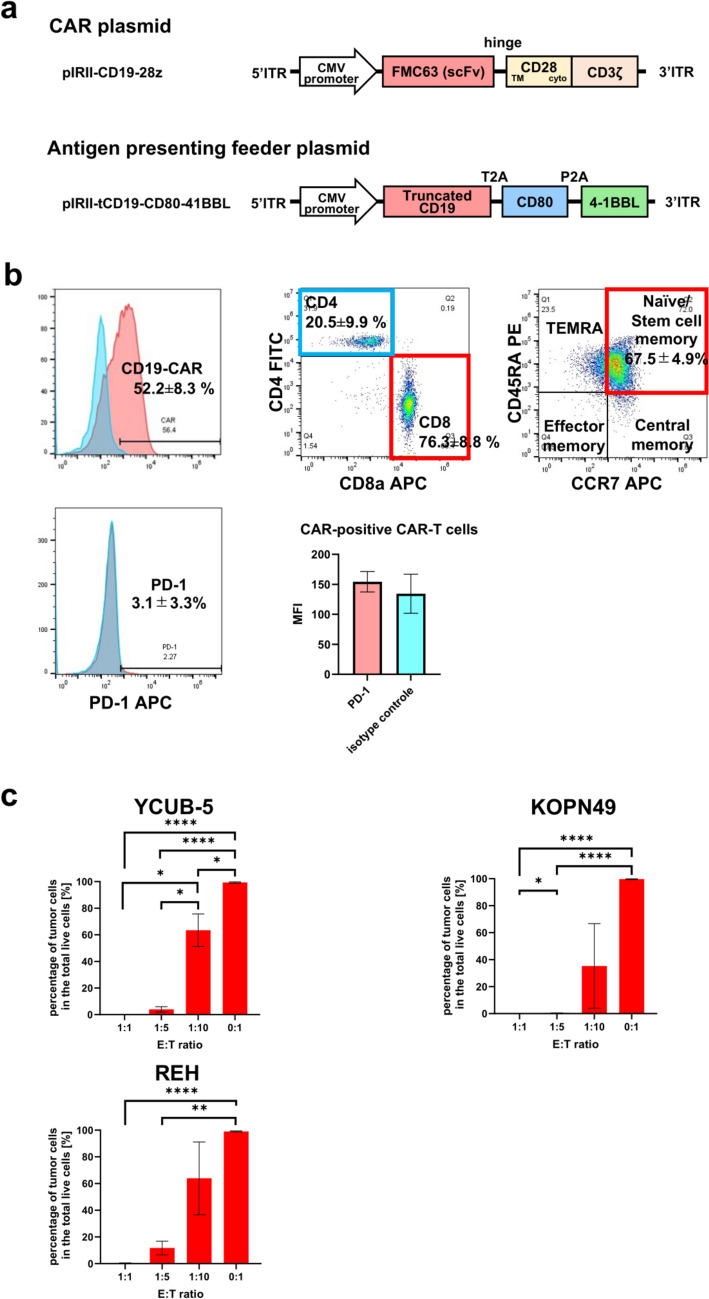
Phenotypic and functional analysis of PiggyBac transposon-mediated CD19 CAR-T cells. **a**. Schematic representation of PB transposon plasmid constructs for CAR-T cells and antigen-presenting feeder cells. "ITR" denotes internal tandem repeat, "TM" indicates the transmembrane domain, and "cyto" represents the cytoplasmic domain. **b**. Representative phenotypes of CD19 CAR-T cells (n = 3). **c**. Anti-leukemic activity of CD19 CAR-T cells against B-ALL cells at various effector-to-target (E:T) ratios. CD19 CAR-T cells and tumor cells were co-cultured for 4 days at different E:T ratios, and the number of live tumor cells was quantified by flow cytometry. Data represent the mean ± SD of three independent experiments

We used the artificially synthesized PB transposase plasmid, pCMV-PB, which contained ~ 2.4 kb of transposase elements with identical 13 base pair (bp) terminal inverted repeats and additional asymmetric 19 bp internal repeats [16] (Mediridge Co., Ltd, Tokyo, Japan). The PB transposon vector encoding the CD19-specific CAR with CD28 and CD3 zeta signaling domains (pIRII-CD19-28z) (Fig. 1a) was constructed as previously described [17]. We utilized antigen-presenting PBMC-derived feeder cells expressing truncated CD19 (tCD19) along with the co-stimulatory molecules CD80 and 4-1BB ligand (CD137L), which were generated by introducing the pIRII-tCD19-CD80-4-1BBL plasmid (Fig. 1a) into autologous PBMC, as described previously [17, 18], for ex vivo expansion of CD19 CAR-transduced T cells.

### Generation of CD19 CAR-T cells

CD19 + CAR-T cells were generated using a PB transposon system [17]. Initially, 4 × 10⁶ freshly isolated PBMCs from healthy volunteers, without prior stimulation, were co-electroporated with 7.5 μg of the pCMV-PB vector and 7.5 μg of the pIRII-CD19-28z transposon in 100 μl of electroporation buffer using the P3 Primary Cell 4D-Nucleofector™ X Kit (Lonza, Program FI-115). Simultaneously, a feeder plasmid, pIRII-tCD19-CD80-4-1BBL, containing genes expressing truncated CD19, CD80, and 4-1BBL, was introduced into 1 × 10^6^ peripheral PBMCs via electroporation to generate antigen-presenting feeder cells. After electroporation, both CAR-T cells and feeder cells were placed in ALyS™705 Medium (Cell Science & Technology Institute, Miyagi, Japan), supplemented with 5% synthetic, animal-free serum (Cell Science & Technology Institute), interleukin-7 (IL-7, 10 ng/ml; Miltenyi Biotec), and interleukin-15 (IL-15, 5 ng/ml; Miltenyi Biotec). Feeder cells were inactivated by ultraviolet irradiation at 24 h post-electroporation and subsequently co-cultured with CAR-T cells for 14 days using established protocols. Control CAR-T cells targeting the EPHB4 receptor were also produced using the same PB transposon system as previously described [19] for comparative analysis in in vivo assays. The study experiments were reported in compliance with the MIATA guidelines [20].

### Flow cytometry

The expression of CD19 CAR molecules on the T cell surface was measured by flow cytometry using recombinant human CD19 Fc chimera protein (R&D Systems, Minneapolis, MN, USA) and goat anti-human immunoglobulin (Ig)-G Fc fragment-specific antibody conjugated to fluorescein isothiocyanate (FITC) (Merck Millipore, Burlington, MA, USA). Allophycocyanin (APC)-conjugated anti-CD3 (RRID:AB_2562045), FITC-conjugated anti-CD19 (RRID:AB_314236), APC-conjugated anti-CD8 (RRID:AB314116), phycoerythrin (PE)-conjugated anti-CD4 (RRID:AB_314076), PE-conjugated anti-CD45RA (RRID:AB_314411), and APC-conjugated anti-CCR7 (RRID:AB_10917387) antibodies (all from BioLegend, San Diego, CA, USA) were used to characterize the phenotypes of CAR-T cells. An APC-conjugated anti-programmed cell death protein-1 (PD-1) antibody (RRID:AB_940475) was used as an exhaustion and senescence marker for CAR-T cells (all from BioLegend). FITC-conjugated anti-CD19 antibody was used to determine the phenotype of leukemic tumor cells. All flow cytometry data were acquired using a BD Accuri™ C6 Plus (BD Biosciences, Franklin Lakes, NJ, USA, RRID:SCR_019591) and analyzed using FlowJo™ software (BD Biosciences, RRID:SCR_008520).

### In vitro* cytotoxicity*

The cytotoxic effects of JAK inhibitors on leukemia cell lines were evaluated by treating the cells with varying concentrations of ruxolitinib, fedratinib, or CHZ868. Cells were incubated in RPMI-1640 medium supplemented with 10% FBS for 4 days, and viable cell counts were determined every 48 h using a Coulter counter (Beckman Coulter, Brea, CA, USA). We established co-cultures with CD19-positive tumor cells at effector-to-target (E:T) ratios of 1:1, 1:5, and 1:10 and a control ratio of 0:1 (tumor cells only) in 24-well plates and incubated them with or without JAK inhibitors to assess CD19 CAR-T cell-mediated cytotoxicity. Four days later, CAR-T cell cytotoxicity was assessed by flow cytometry, and the percentage of live tumor cells was determined by calculating the ratio of CD19-positive and CD3-negative cells relative to the total number of live cells. B-ALL and CD19 CAR-T cells were co-cultured with 100 nM of each JAK inhibitor at an appropriate E:T ratio to confirm the effect of each JAK inhibitor on the cytotoxicity of CD19 CAR-T cells.

### Sequential killing assay

A dynamic assessment of the cytotoxicity of CD19 CAR-T cells was performed using a sequential killing assay. Initially, 1.5 × 10^5^ REH cells and 7.5 × 10^4^ CAR-T cells, and 3.0 × 10^5^ KOPN-49 cells with 1.5 × 10^5^ CAR-T cells were co-cultured in 24-well plates with or without JAK inhibitors. The cytotoxic activity of CAR-T cells was evaluated by flow cytometric analysis of residual tumor cells 4 days after co-culture. Subsequently, fresh tumor cells (1.5 × 10^5^ for REH and 3.0 × 10^5^ for KOPN-49, irrespective of CAR-T cell expansion in each well) were added to the CAR-T cells, and the process was repeated every 4 days for a total of 3 cycles to monitor sustained cytotoxicity. Simultaneously, CAR-T cell proliferation was assessed using flow cytometry to evaluate their expansion in response to tumor cell rechallenge.

### Cytokine production assay

Concomitant with the sequential killing assay described above, cytokine release was measured to gauge the functional response of CAR-T cells to multiple tumor cell challenges. Culture supernatants were collected 24 h after each rechallenge with tumor cells and analyzed for IFN-γ, TNF, and IL-2 levels using a Cytometric Bead Array kit (BD Biosciences). Data acquisition and cytokine quantification were performed using a BD Accuri C6 Plus flow cytometer and FCAP Array Software v.3.0, respectively (both from BD Biosciences).

### RNA transcriptome analysis

Surviving CAR-T cells were isolated following two sequential co-cultures with REH cells at an E:T ratio of 1:2, with and without CHZ868. To ensure sample purity, flow cytometry was used to identify CAR-T cells, confirming the presence of only CD19- CD3 + cells, with no CD19 + CD3- cells detected. Total RNA was extracted from the enriched CAR-T cell populations using the RNeasy Minikit (QIAGEN, Hilden, Germany) and subjected to rigorous quality assessment and quantification with the NanoDrop™ Lite Spectrophotometer (Thermo Fisher Scientific Inc., RRID:SCR_025369) before high-throughput RNA sequencing. Transcriptome analysis was outsourced to Macrogen JAPAN (Tokyo, Japan), where RNA integrity was further verified, and libraries were prepared for sequencing. The resulting sequencing data were used to analyze the gene expression profiles of CAR-T cells under the influence of CHZ868 to investigate the molecular mechanisms underlying CAR-T cell function and persistence.

### Quantitative real-time PCR analysis

After sequential tumor rechallenges, total RNA was extracted from CAR-T cells using the RNeasy Mini Kit (QIAGEN, Hilden, Germany). Complementary DNA (cDNA) was synthesized from the extracted RNA using the SuperScript™ VILO™ cDNA Synthesis Kit (Thermo Fisher Scientific Inc.). Quantitative real-time PCR was performed on the 7500 Fast Real-Time PCR System (Applied Biosystems, Waltham, MA, USA) with TB Green dye (Takara Bio, Shiga, Japan). Cycle threshold (Ct) values for each target mRNA and the housekeeping gene glucose-6-phosphate dehydrogenase (GAPDH) were determined, with GAPDH serving as the reference gene for normalization. Relative expression levels of target mRNAs were calculated using the ΔCt method, normalized to GAPDH expression. Primer sequences used in this analysis are indicated in Supplementary Table 2.

### In vivo* experiments*

Six-week-old female NOD.Cg-Prkdc^scid^Il2rg^tm1Wjl^/SzJ (NSG) mice (RRID:IMSR_JAX:005557) were purchased from Jackson Laboratory (Bar Harbor, ME, USA) and housed at the Kyoto Prefectural University of Medicine for over a week before the start of the experiments, with free access to food and water. The mice were intravenously injected with 5 × 10^5^ REH pIRII-FFLuc-puroR-GFP cells via the tail vein to establish leukemia. After tumor engraftment, confirmed seven days post-inoculation, the mice received an intravenous infusion of either 1 × 10^5^ CD19 CAR-T cells or EPHB4 CAR-T cells. Concurrently, CHZ868 (30 mg/kg/day) or vehicle control (DMSO) was administered orally over a three-week period. The tumor burden was monitored non-invasively through bioluminescent imaging using the IVIS Lumina Series III system (PerkinElmer Inc., Waltham, MA, USA, RRID:SCR_025239). Quantitative analysis of tumor growth was performed by measuring photon emissions from the regions of interest using Living Image software v2 (PerkinElmer, Inc.). Bone marrow samples were periodically collected by aspiration from the tibias to assess the persistence of human T cells in vivo. These samples were stained with an APC-conjugated anti-human CD3 antibody (BioLegend) and analyzed using flow cytometry to determine the long-term engraftment of human T cells. The humane endpoint of the experiment was determined in accordance with the guidelines provided by the Center for Comparative Medicine at Kyoto Prefectural University of Medicine, and the mice were euthanized accordingly.

### Statistical analysis

Statistical comparisons between groups were performed using two-tailed parametric or non-parametric tests (Mann–Whitney U-test) for unpaired data, two-tailed paired Welch’s t-tests for matched samples. All data are presented as the mean ± standard deviation. The log-rank test was used to compare the survival curves obtained using the Kaplan–Meier method. Statistical significance was set at *P* < 0.05. All statistical analyses were performed using Prism 10 software (GraphPad Software, San Diego, CA, USA, RRID:SCR_002798).

## Results

### Generation and phenotype of CD19 CAR-T cells

We successfully generated CD19 CAR-T cells using PB transposon-mediated gene transfer and achieved 52.2 ± 8.3% CAR positivity (Fig. 1b). These CAR-T cells displayed a predominance of CD8 + T cells, with 20.5 ± 9.9% CD4 + and 76.3 ± 8.8% CD8 + cell populations (Fig. 1b). Notably, CD19 CAR-T cells primarily exhibited the CD45RA + /CCR7 + phenotype characteristic of stem cell memory T cells (67.5 ± 4.9%), which are known for their potent and durable anti-tumor efficacy (Fig. 1b). Additionally, these cells showed minimal expression of the exhaustion marker Programmed Death-1 (PD-1) (3.1 ± 3.3%) without an increase in the mean fluorescence intensity, even after extensive proliferation (Fig. 1b, Supplementary Fig. 1). Consistent with previous findings [17], CD19 CAR-T cells demonstrated strong and sustained cytotoxicity against CD19 + leukemic cells in a dose-dependent manner (Fig. 1c, Supplementary Fig. 2).

### Impact of JAK pathway inhibition on B-ALL cells based on JAK2 mutation status

We treated three B-ALL cell lines with ruxolitinib (a JAK1/JAK2 inhibitor), fedratinib (a type I selective JAK2 inhibitor), and CHZ868 (a type II selective JAK2 inhibitor) to evaluate the effect of JAK inhibitors on B-ALL cell lines with varying JAK2 mutation statuses. The half-maximal inhibitory concentration (IC_50_) was calculated to determine inhibitor efficacy (Supplementary Table 1). Despite the presence of JAK2 mutations, the YCAB-5 and KOPN-49 cell lines exhibited resistance to ruxolitinib, likely due to additional KRAS mutations [21]. Conversely, YCAB-5 and KOPN-49 cells were more sensitive to the type I JAK2 inhibitor fedratinib and the type II JAK2 inhibitor CHZ868 than to ruxolitinib. The JAK2 wild-type REH cell line was resistant to all the tested JAK pathway inhibitors (Supplementary Table 1).

### *CHZ868 enhances the cytotoxic effect of CD19 CAR-T cells *in vitro

**Fig. 2:**
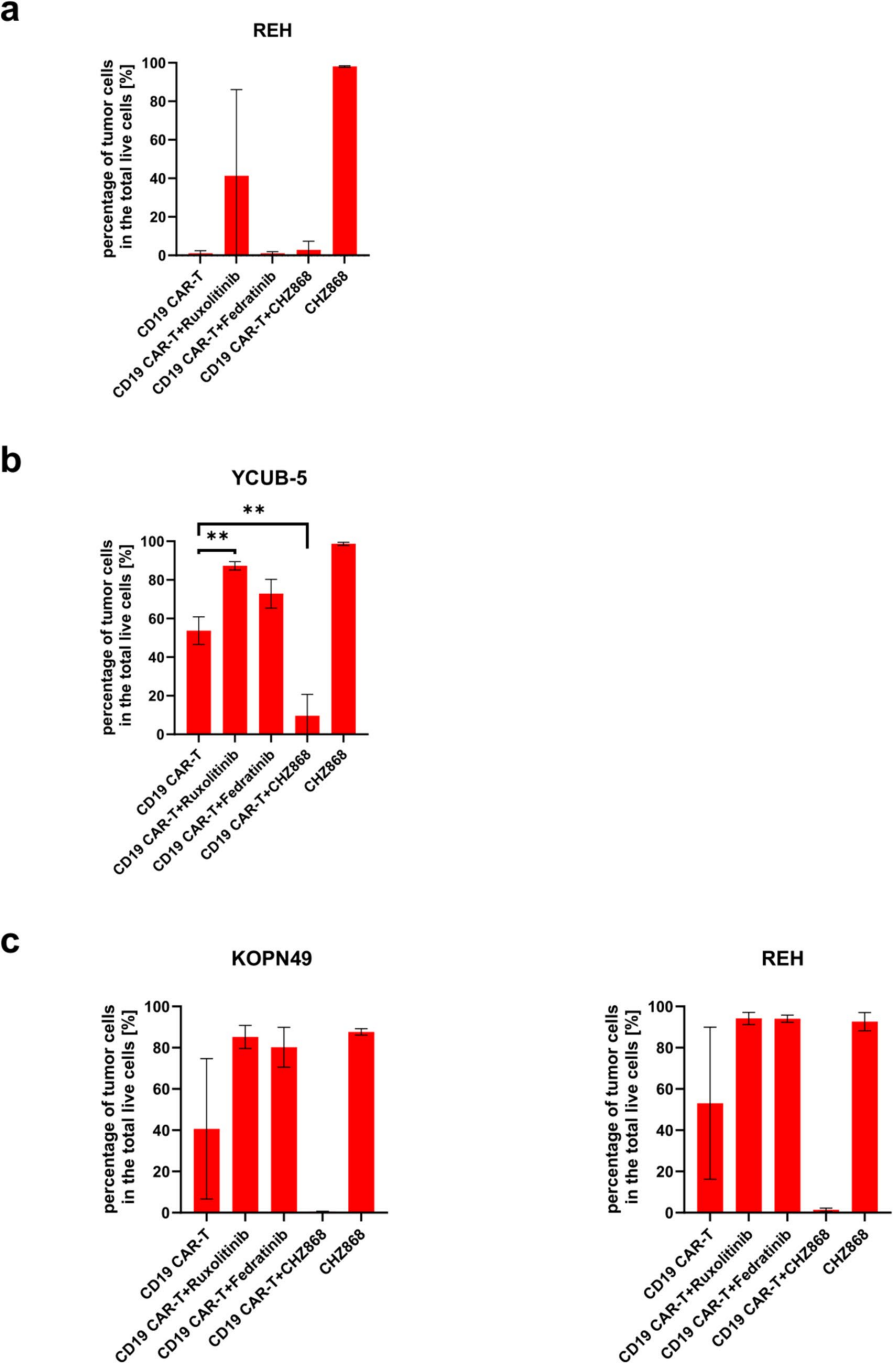
Effects of combinatorial treatment of CD19 CAR-T cells with JAK inhibitors on B-ALL cells. **a**. REH cells were co-cultured with CD19 CAR-T cells at an E:T ratio of 1:2 and treated with DMSO, ruxolitinib, fedratinib, or CHZ868. After 4 days, the numbers of live tumor and CAR-T cells were quantified by flow cytometry. **b**. YCUB-5 cells were co-cultured with CD19 CAR-T cells at an E:T ratio of 1:7.5, with treatments as above. The ratio of live tumor cells to live CAR-T cells, along with standard deviations, was assessed by flow cytometry after 4 days (n = 3). **c** KOPN-49 and REH cells were sequentially co-cultured with CD19 CAR-T cells over three cycles, each lasting 4 days. Fresh tumor cells (1.5 × 10^5^ for REH and 3.0 × 10^5^ for KOPN-49), irrespective of CAR-T cell expansion in each well) were added at each cycle to evaluate sustained cytotoxicity (n = 3)

To evaluate the impact of JAK inhibitors on the tumoricidal activity of CD19 CAR-T cells, REH cells were co-cultured with CD19 CAR-T cells at an E:T ratios 1:2 in the presence of ruxolitinib, fedratinib, or CHZ868. Consistent with previous findings, 100 nM ruxolitinib blocked the cytotoxic effects of CD19 CAR-T cells on JAK2 wild-type REH cells (Fig. 2a, Supplementary Fig. 3b). In contrast, selective JAK2 inhibitors, namely, fedratinib and CHZ868, did not compromise the efficacy of CD19 CAR-T cells, allowing CAR-T cells to effectively eliminate REH cells in the presence of these inhibitors, comparable to that observed in the CAR-T cell monotherapy group (Fig. 2a, Supplementary Fig. 3b).

Given the strong anti-tumor efficacy of CD19 CAR-T cells in REH cells, we hypothesized that a more challenging model would be suitable for evaluating the effects of JAK2 inhibitors on CD19 CAR-T cells. Therefore, we co-cultured CD19 CAR-T cells with YCUB-5 cells at an E:T ratio of 1:7.5, a condition under which CD19 CAR-T cells alone cannot eradicate YCUB-5 cells (Fig. 1c). As single agents, neither CD19 CAR-T cells nor selective JAK2 inhibitors (fedratinib and CHZ868) controlled YCUB-5 cell growth. However, the combination of CD19 + CAR-T cells and CHZ868 eradicated the YCUB-5 cells (Fig. 2b, Supplementary Fig. 3a). For KOPN-49 and REH cells, we performed a tumor rechallenge assay in which fresh tumor cells were added to CAR-T cells every three days in the presence of selective JAK2 inhibitors. This assay simulates a clinical setting in which a single population of CAR-T cells repeatedly kills tumor cells to eradicate the tumor. CD19 CAR-T cells effectively killed KOPN-49 and REH cells during the first tumor challenge but failed to control tumor growth in the third round, likely due to T cell exhaustion induced by multiple antigen stimulation (Fig. 2c, Supplementary Fig. 3b). Ruxolitinib completely impaired the anti-tumor function of CD19 CAR-T cells during three rounds of tumor rechallenge (Fig. 2c, Supplementary Fig. 3b). Interestingly, in the presence of fedratinib and CHZ868, CD19 CAR-T cells maintained their anti-tumor function even in the multiple rounds of tumor rechallenge, including JAK2 wild-type REH cells (Fig. 2c, Supplementary Fig. 3b). CHZ868 demonstrated a greater synergistic effect on CD19 CAR-T cells than fedratinib in both KOPN-49 and REH cells.

**Fig. 3:**
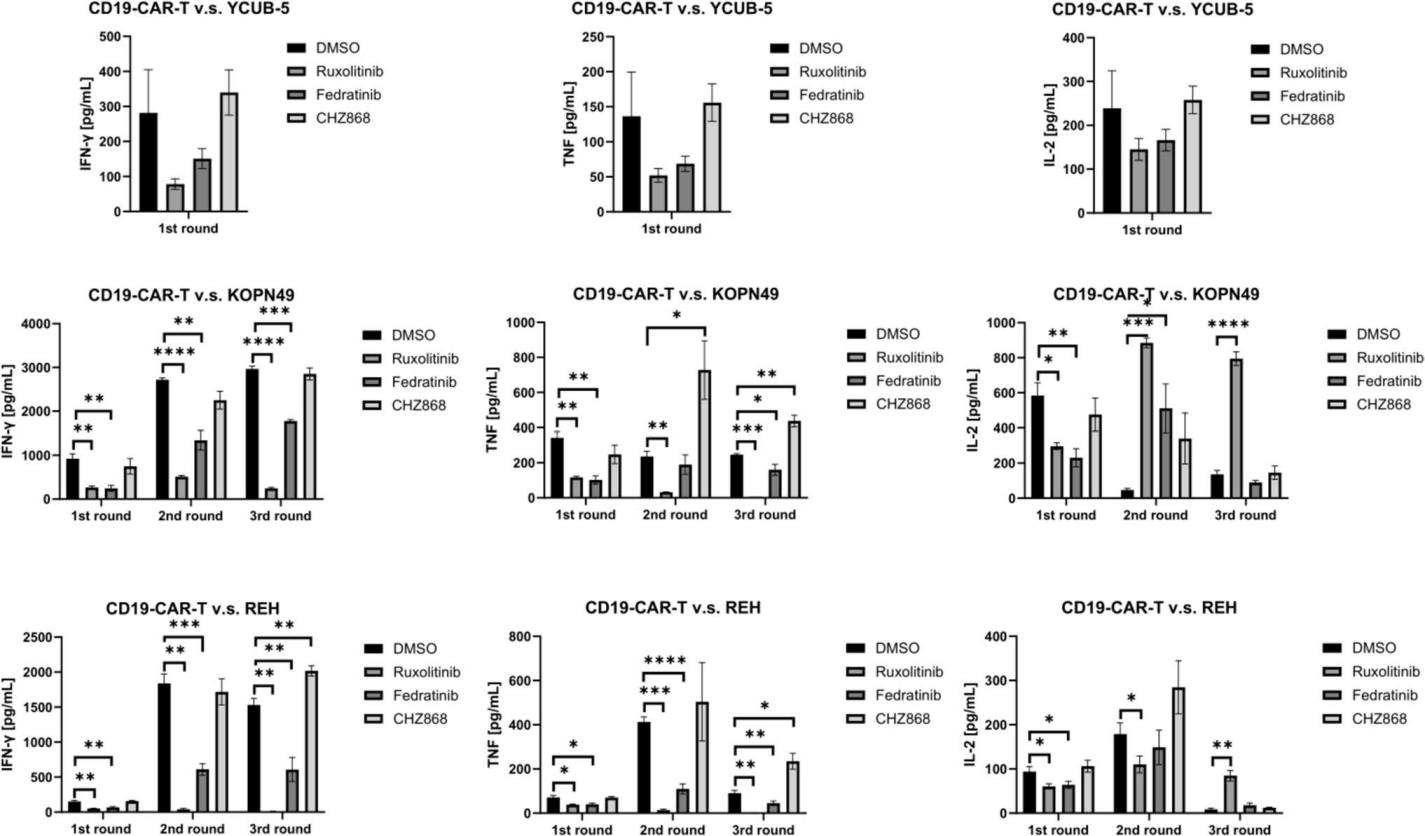
Cytokine release by CD19 CAR-T cells during sequential tumor rechallenge. Levels of IFN-γ and TNF in the supernatant from co-cultures of CAR-T cells with B-ALL cells were measured following sequential co-cultures (n = 3). Data are presented as mean ± standard deviation, with significance indicated by *p < 0.05, **p < 0.01, ***p < 0.001, ****, and *p* < 0.0001

To further investigate the synergistic effect of JAK2 pathway inhibition on the anti-tumor activity of CD19 CAR-T cells, we measured cytokine levels following co-culture. IFN-γ, TNF, and IL-2 levels were significantly reduced when YCUB-5 cells were co-cultured with CD19 CAR-T cells in the presence of ruxolitinib, indicating impaired cytotoxicity (Fig. 3). However, cytokine levels were maintained when CD19 CAR-T cells were co-cultured with CHZ868 cells, similar to those in the control group (Fig. 3). In the KOPN-49 and REH experiments, where CHZ868 demonstrated synergistic effects in the tumor rechallenge test, IFN-γ and TNF secretion was also maintained during the third tumor rechallenge, even in REH cells with wild-type JAK2 (Fig. 3). These data suggest that CHZ868 enhances the anti-tumor function of CD19 CAR-T cells, regardless of the JAK2 mutation status of the tumor cells.

### CHZ868 maintained the memory phenotype of CD19 CAR-T cells after antigen stimulation

**Fig. 4:**
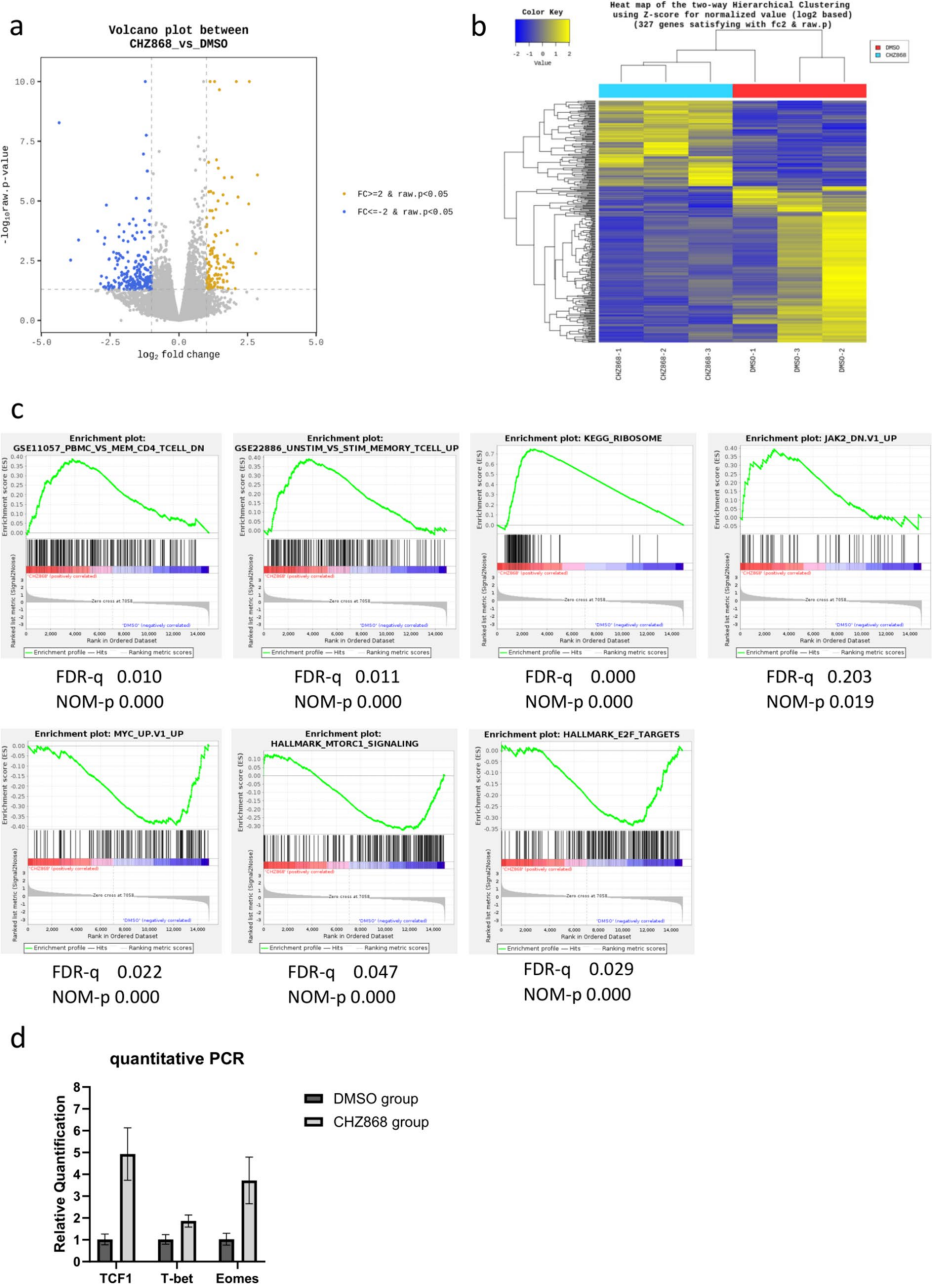
Transcriptomic profiling of CHZ868-treated CD19 CAR-T cells. **a**. Volcano plot displaying genes significantly upregulated or downregulated in CHZ868-treated CAR-T cells compared to control, with adjusted FDR < 0.05. **b**. Heatmap showing two-way hierarchical clustering of genes, based on Z-score normalization of expression values (Log2 scale). **c**. Gene sets differentially enriched in CHZ868-treated versus control groups, with nominal p-values < 5%. **d**. Relative quantification of key transcription factors involved in T cell differentiation and exhaustion (TCF-1, T-bet, EOMES) by quantitative PCR

Given the synergistic effect of CHZ868 on CD19 CAR-T cells in JAK2 wild-type REH cells, we hypothesized that CHZ868 directly enhanced the anti-tumor function of CD19 CAR-T cells by modulating their characteristics during tumor interactions. To test this hypothesis, we monitored phenotypic changes in CD19 CAR-T cells, specifically examining the expression of CD45RA and CCR7 using flow cytometry across three sequential tumor rechallenges. However, no significant phenotypic differences were observed between the DMSO and CHZ868 groups following these co-cultures (Supplementary Fig. 4a, 4b). Therefore, to investigate this at the molecular level, we collected CD19 CAR-T cells after two consecutive co-cultures with REH cells in the presence of either DMSO (control) or CHZ868 and performed transcriptome analysis. We identified 116 genes with higher expression levels and 211 genes with lower expression levels in the CHZ868 group than in the control group (Fig. 4a, b). Gene Set Enrichment Analysis (GSEA) revealed decreased expression of activation-related genes, such as MYC and mTOR, and increased expression of genes related to inactive memory T cells in the CHZ868 group (Fig. 4c). Ribosome-related genes, which reflect proliferative ability in early differentiation stages, were upregulated in the CHZ868 group (Fig. 4c). These data suggest that CHZ868 maintained the memory T cell fraction and proliferative ability despite multiple tumor rechallenges, which likely contributed to the persistence of the overall potency of CD19 CAR-T cells. To further explore transcriptional regulation, we examined the expression of key transcription factors associated with T cell differentiation and exhaustion (TCF-1, T-bet, and Eomes) via quantitative PCR. T-bet, which is typically associated with effector differentiation, showed mild upregulation, whereas TCF-1, which is highly expressed in naïve and memory T cells, and Eomes, a critical factor in central memory T cell formation [22], were notably elevated in the CHZ868 group (Fig. 4d). These results suggest that CHZ868 promotes the memory phenotype of CAR-T cells, thereby preserving their anti-leukemic activity.

### *CHZ868 enhances the anti-tumor effect of CD19 CAR-T cells *in vivo

**Fig. 5:**
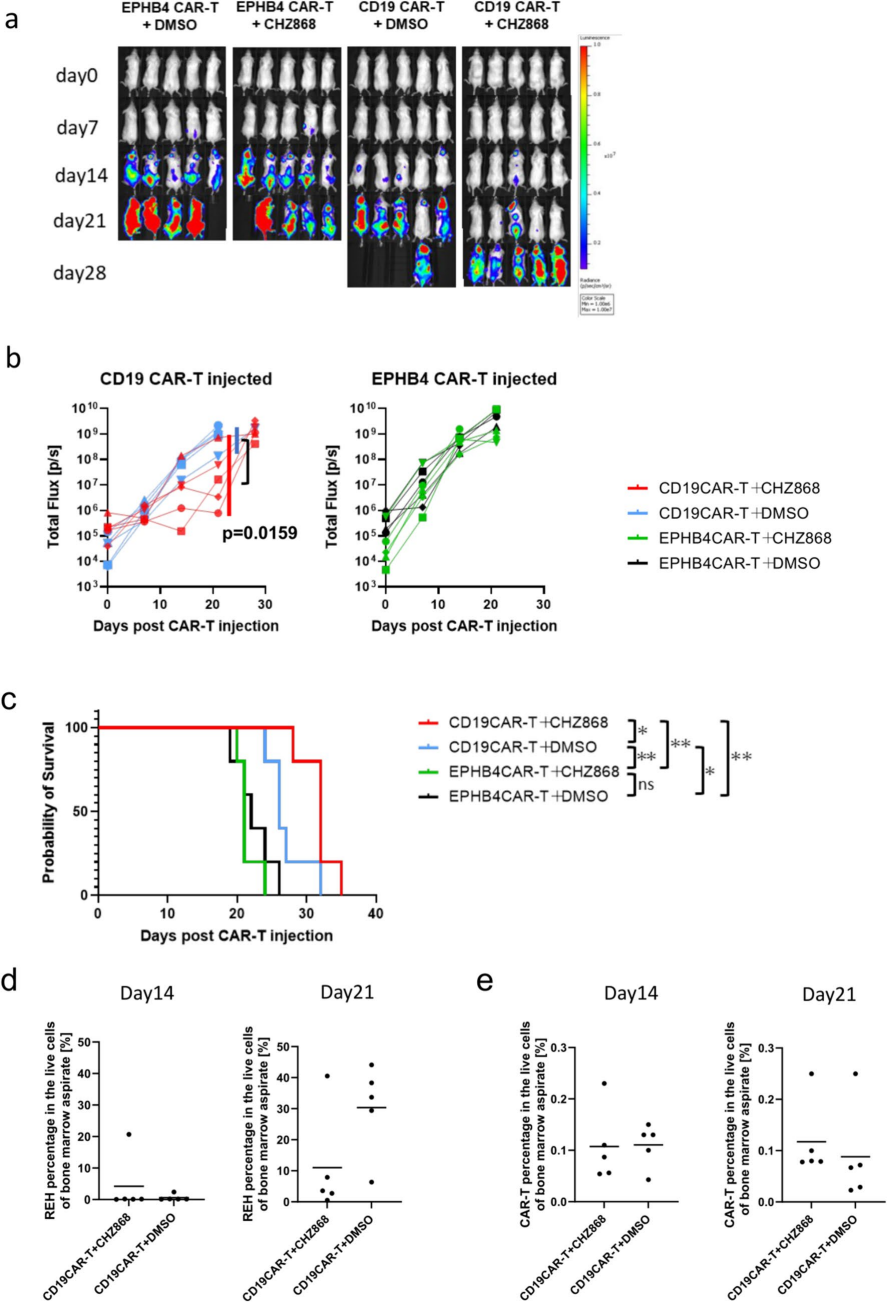
Combinatorial treatment with CHZ868 enhances survival in JAK2-wild-type REH bearing mice. **a**. Bioluminescence imaging of mice following intravenous infusion of CAR-T cells (n = 5). **b**. Tumor volumes measured by total flux (photons per second). **c**. Overall survival rates post-CAR-T cell infusion, analyzed by log-rank test (ns *p* > 0.05, * *p* < 0.05, ** *p* < 0.01). **d**. Percentage of live tumor cells and **e**. CAR-T cells in the bone marrow on day 14 and 21

We conducted an in vivo stress test wherein the CAR-T cell dosage was reduced to functional limits, requiring these cells to persist without immune exhaustion to eradicate tumor cells to confirm the synergistic effect of CHZ868 on CD19 CAR-T cells in vivo. In the control group treated with EPHB4 CAR-T cells, no anti-leukemic effect was observed and CHZ868 monotherapy did not exhibit specific cytotoxicity. In contrast, the CD19 CAR-T cell group showed a modest anti-leukemic effect, which was enhanced in combination with CHZ868, as evidenced by live tumor imaging (Fig. 5a, b), prolonged survival (Fig. 5c), and the number of viable tumor cells in the bone marrow on day 21 (Fig. 5d). There was no statistically significant difference in the number of live CAR-T cells in the bone marrow between the CD19 CAR-T cell and CD19 CAR-T cell/CHZ868 groups (Fig. 5e). For the safety assessment, no significant phenotypic changes associated with CAR-T cell-based toxicities were observed, including body-weight loss in the mice during the experiment (Supplementary Fig. 5).

## Discussion

In this study, we evaluated the combinatorial treatment of selective JAK2 inhibitors and CD19 CAR-T cells in both JAK2 mutant and wild-type B-ALL. The type II selective JAK2 inhibitor, CHZ868, did not compromise the anti-tumor efficacy of CD19 CAR-T cells. Instead, CHZ868 synergized with the potency of CD19 CAR-T cells by maintaining their memory phenotype. These synergistic effects were also observed in vivo, where the combination of CD19 CAR-T cells and CHZ868 prolonged the survival of JAK2 wild-type B-ALL model mice compared to CD19 CAR-T cell monotherapy. Thus, selective inhibition of the JAK2 pathway improves the function of CD19 CAR-T cells against B-ALL, regardless of the JAK2 mutation status, which holds promise for improving the prognosis of refractory B-ALL.

The combination of CAR-T cells with molecular-targeting agents has been extensively investigated to enhance the anti-tumor efficacy of these therapies. These strategies aim for direct tumor cell targeting and synergistically enhancing CAR-T cell function. The prevention of CAR-T cell exhaustion by modulating the PD-L1/PD-1 axis with anti-PD-L1 or PD-1 antibodies has shown promise in preclinical studies and clinical trials [23–26]. Another approach using dasatinib [9] and trametinib [10] with GD2 CAR-T cells demonstrated increased anti-tumor activity by reducing overactivation and subsequent exhaustion of GD2 CAR-T cells, as these drugs modulate T cell receptor (TCR) activation through the inhibition of Lck or the Raf/MEK pathway. Similarly, the commercially available JAK1/2 inhibitor, ruxolitinib, was previously applied to CD19 CAR-T cells against JAK-mutant B-ALL to achieve an additive effect on tumor cells and CD19-directed immunotherapy [11]. However, ruxolitinib completely blocked the potency of CD19 CAR-T cells, as it strongly affected JAK1 and JAK3, which are crucial for TCR activation [15]. Given that JAK2 is not involved in TCR signaling, but downstream of cytokine receptors, including IL-6, we hypothesized that selective JAK2 inhibition would not compromise CAR-T cell function. As expected, fedratinib and CHZ868 did not diminish the anti-tumor effect of CAR-T cells, likely due to their minimal effects on JAK1/3. Thus, combinatorial treatment with selective JAK2 inhibitors and CD19 CAR-T cells represents an attractive therapeutic option for JAK2 mutant Ph-like ALL, which is an aggressive B-ALL subtype resistant to standard chemotherapies that often acquires JAK2 mutations.

Notably, CHZ868 demonstrated synergistic anti-tumor effects with CD19 CAR-T cells, even in JAK2 wild-type B-ALL cells. Comprehensive transcriptome analysis revealed that CHZ868 suppressed T cell activation signals and maintained a memory T cell phenotype with a high proliferative capacity after multiple tumor rechallenges, suggesting that CHZ868 might prevent T cell differentiation and exhaustion by antigen stimulation. In particular, our data show that in the CHZ868-treated group, TCF-1, Eomes, and T-bet expression levels were elevated compared to those in controls. TCF-1, which is crucial for early T cell differentiation and the maintenance of stem-like qualities, maintained robust expression even after repeated antigen exposure. This aligns with its established role in supporting self-renewal and proliferative potential in naïve and central memory T cells, which are favorable in CAR-T therapies for their durability and capacity for long-term immunity [27]. Conversely, Eomes and T-bet—transcription factors associated with T cell differentiation into memory and effector phenotypes—were modestly induced in the CHZ868 group. Eomes supports long-term memory potential, while T-bet facilitates effector functions and cytotoxicity [28]. This controlled expression pattern suggests that CHZ868 prevents terminal differentiation, instead promoting a progenitor-like, less exhausted T cell phenotype. Recent studies indicate that TCF-1 marks a subset of progenitor-like exhausted T cells with self-renewal capacity and potential for functional recovery through checkpoint inhibition, such as PD-1 blockade [29]. Our findings suggest that CHZ868 promotes a similar progenitor-like state, preserving a less differentiated CAR-T cell subset with sustained anti-tumor efficacy even under chronic antigen exposure. Additionally, maintaining the memory T cell fraction while suppressing overactivation by CHZ868 preserved the overall anti-tumor efficacy of CD19 CAR-T cells. Similar to CHZ868, the selective JAK2 inhibitor, ZT55, suppressed CD25 expression in naïve T cells [30]. CD25, also known as IL-2 receptor alpha chain, is necessary for the formation of high-affinity IL-2 receptors. Reduced expression of this receptor may decrease T cell sensitivity to IL-2. Although IL-2 stimulates T cell proliferation and activation, it is less effective than IL-7 and IL-15 in maintaining stem cell-like memory T cells and may induce early CAR-T cell exhaustion [31]. Given that IL-2 secretion did not differ between the CHZ868 and control groups after sequential co-culture, the enhancement and maintenance of anti-tumor activity in this study was likely due to the modulation of cytokine signals other than IL-2.

The synergistic effect of CHZ868 and CD19 CAR-T cell therapy was more prominent than that of fedratinib. Type I inhibitors bind to the ATP-binding site in the active conformation of JAK2, whereas type II inhibitors bind to both the ATP-binding site and an adjacent allosteric site, stabilizing the inactive conformation of JAK2 and making it less likely to become active [4]. Whereas, type II inhibitors often exhibit higher selectivity because the inactive conformation and allosteric sites are less conserved among different kinases than the ATP-binding site [4, 32], which explains the superior synergistic effect of CHZ868 on CD19 CAR-T cells.

CHZ868 is primarily used to treat JAK2 mutant myeloproliferative neoplasms and B-ALL [4, 33]. However, our study demonstrated enhanced anti-tumor effects in REH cells, which lack JAK2 mutations and were unaffected by CHZ868 monotherapy when combined with CD19 CAR-T cells. This suggests that the combination of CHZ868 and CAR-T cells can produce synergistic effects regardless of the genetic background of the tumor. Therefore, CHZ868 has the potential to enhance CAR-T cell anti-tumor efficacy not only in JAK2-related tumors, but also in a broader range of malignancies.

This study has some limitations, such as the lack of detailed mechanistic insights into how selective JAK2 pathway inhibition affects T cell activation and exhaustion. Further in-depth analyses of intracellular signaling pathways are needed to clarify these mechanisms. Additionally, we cannot rule out the possibility that the observed effects stem from factors other than kinase inhibition. To confirm that our results are indeed due to JAK2 inhibition, future experiments using JAK2 knockdown CAR-T cells should be considered. Unfortunately, we were unable to access patient samples and PDX models, which limited our ability to perform certain experiments. Furthermore, we did not explore alternative CAR constructs, such as those utilizing the 4-1BB co-stimulatory domain. Moreover, exploring the potential synergistic effect of selective JAK2 inhibition in other tumor models, including solid tumors, would be attractive because CAR-T cell therapy for solid tumors is challenging and multiple efforts are being made to improve CAR-T cell immune exhaustion in the tumor microenvironment.

In conclusion, the combination of CHZ868 and CD19 CAR-T cells is a promising strategy for enhancing the efficacy of CAR-T cell therapy without inducing T cell exhaustion. This combination therapy has the potential to improve treatment outcomes in patients with B-ALL and other malignancies, warranting further clinical investigation.

## Supplementary Information

Below is the link to the electronic supplementary material.

Supplementary file1

Supplementary file2

Supplementary file3

Supplementary file4

Supplementary file5

Supplementary file6

Supplementary file7

## Data Availability

The datasets generated during and/or analyzed during the current study are available from the corresponding author upon reasonable request.

## Abbreviations

APC: Allophycocyanin
B-ALL: B-cell acute lymphoblastic leukemia
bp: Base pair
CAR-T: Chimeric antigen receptor T
CAR: Chimeric antigen receptor
cDNA: Complementary DNA
CRS: Cytokine release syndrome
Ct: Cycle threshold
D-PBS: Dulbecco’s phosphate-buffered saline
E:T: Effector-to-target
FACS: Fluorescence-activated cell sorting
FBS: Fetal bovine serum
FFLuc: Firefly luciferase
GAPDH: Glucose-6-phosphate dehydrogenase
GFP: Green fluorescent protein
GSEA: Gene set enrichment analysis
Ig: Immunoglobulin
IL: Interleukin
JAK: Janus kinase
PB: PiggyBac
PBMCs: Peripheral blood mononuclear cells
PD-1: Programmed cell death protein-1
PE: Phycoerythrin
Ph-like ALL: Philadelphia chromosome-like acute lymphoblastic leukemia
Ph +: Philadelphia chromosome positive
tCD19: Truncated CD19
TCR: T cell receptor
TKIs: Tyrosine kinase inhibitors
