# Supplementary figures and images for: Selective JAK2 pathway inhibition enhances anti-leukemic functionality in CD19 CAR-T cells
Source: Cancer Immunol Immunother. 2025 Feb 1;74(3):79. doi: 10.1007/s00262-024-03927-8 (PMC11787079; doi:10.1007/s00262-024-03927-8)

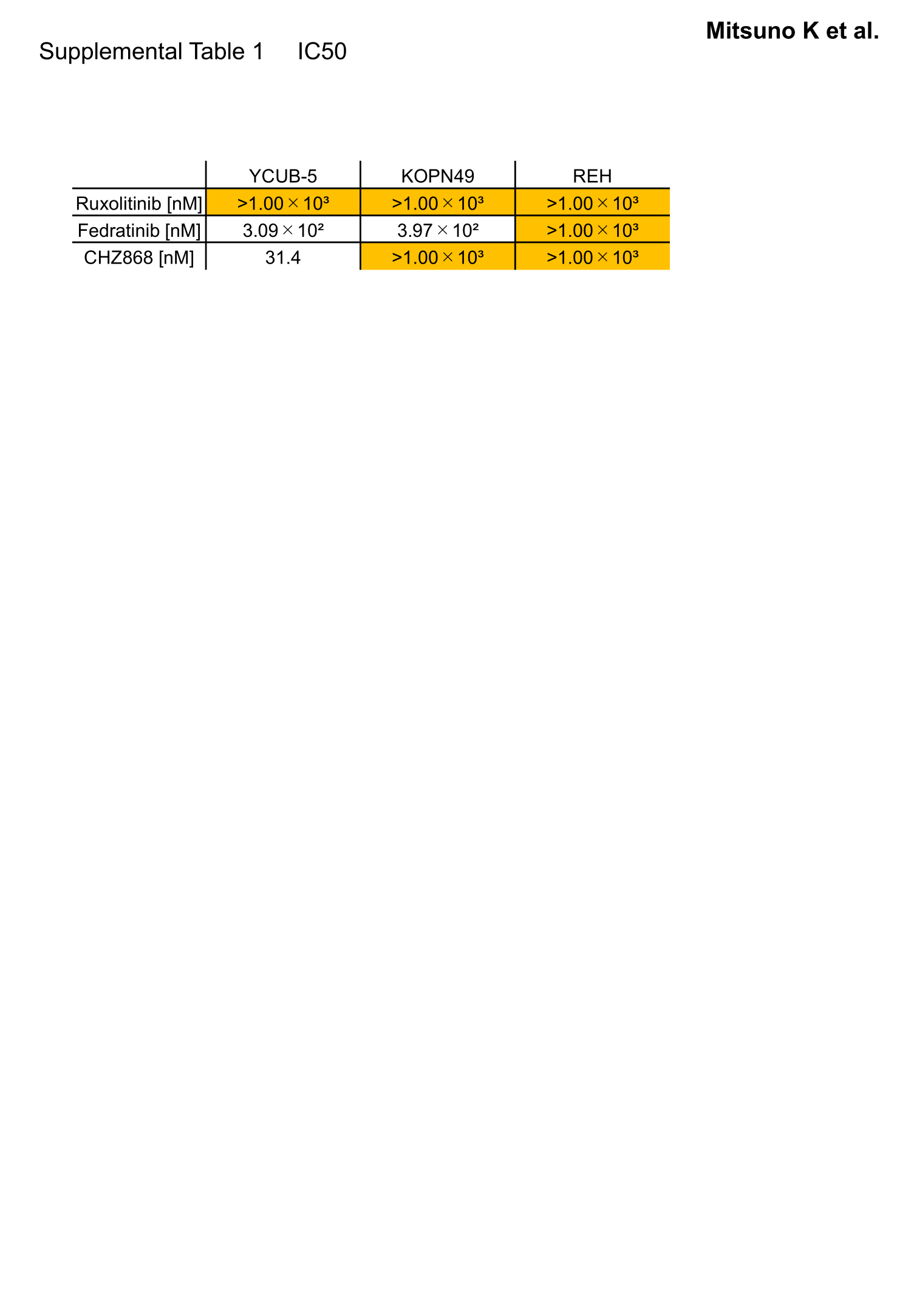

Supplement: Supplementary file 1 — Supplementary file1 [file 262_2024_3927_MOESM1_ESM.tiff]

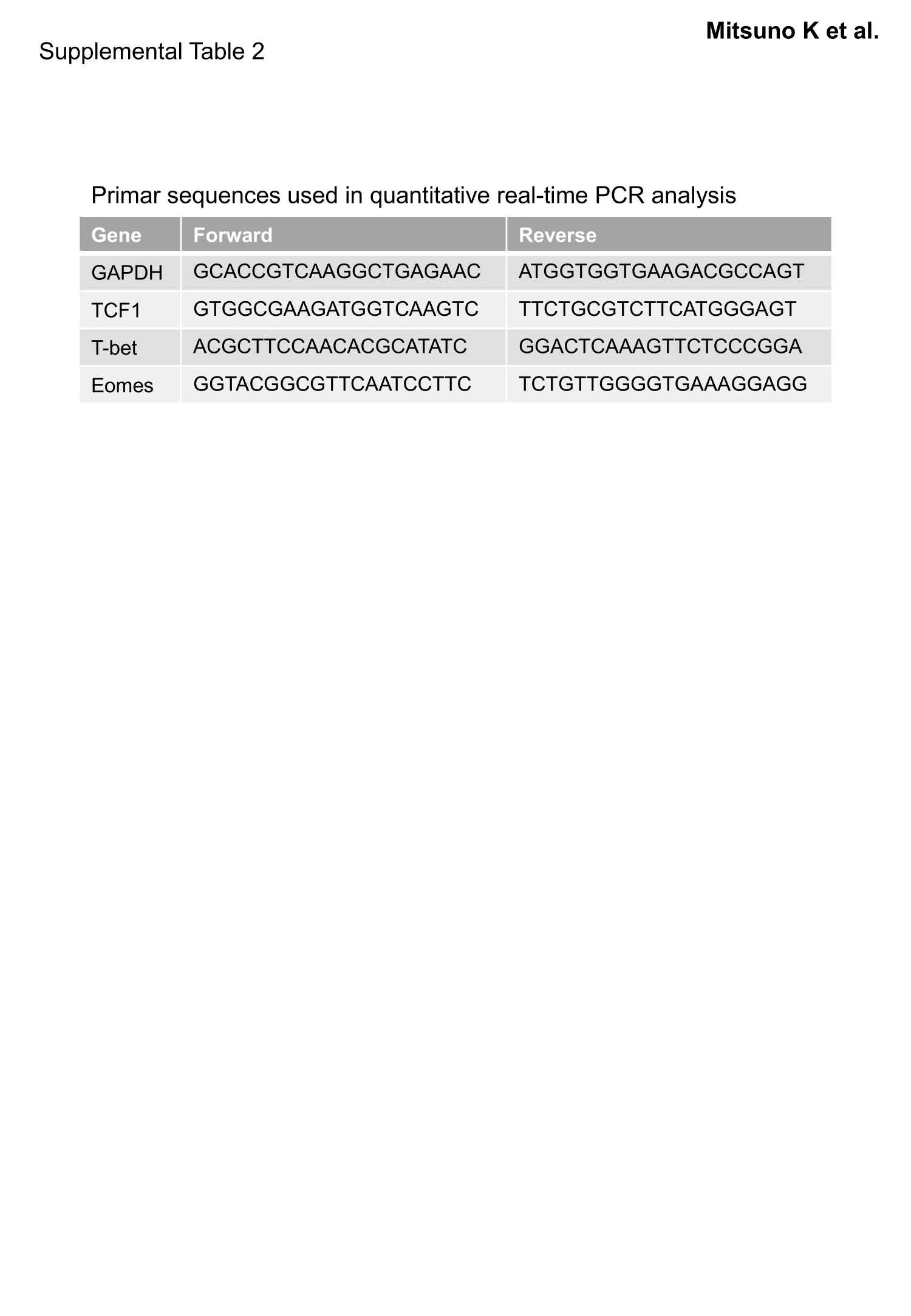

Supplement: Supplementary file 2 — Supplementary file2 [file 262_2024_3927_MOESM2_ESM.tiff]

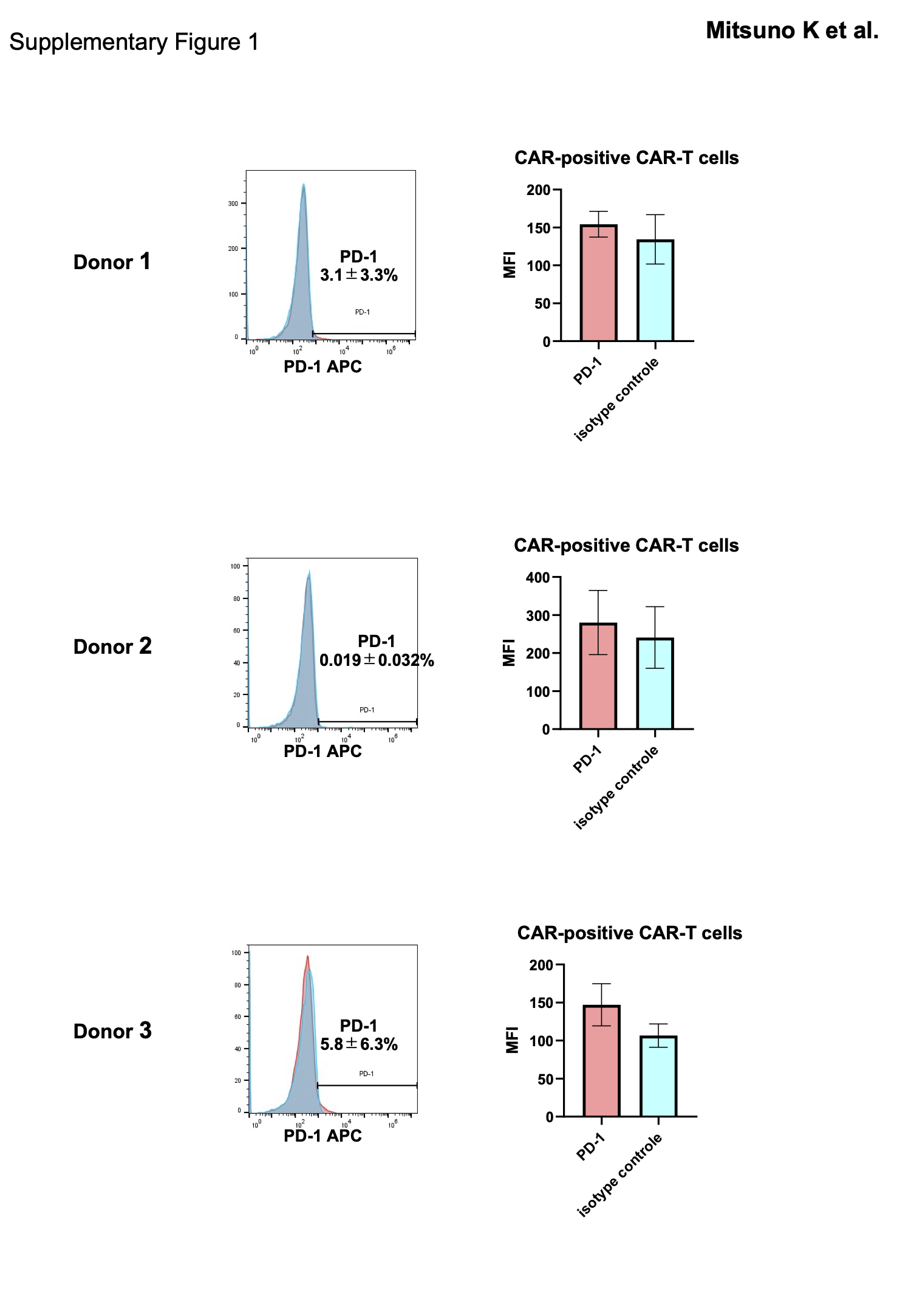

Supplement: Supplementary file 3 — Supplementary file3 [file 262_2024_3927_MOESM3_ESM.tiff]

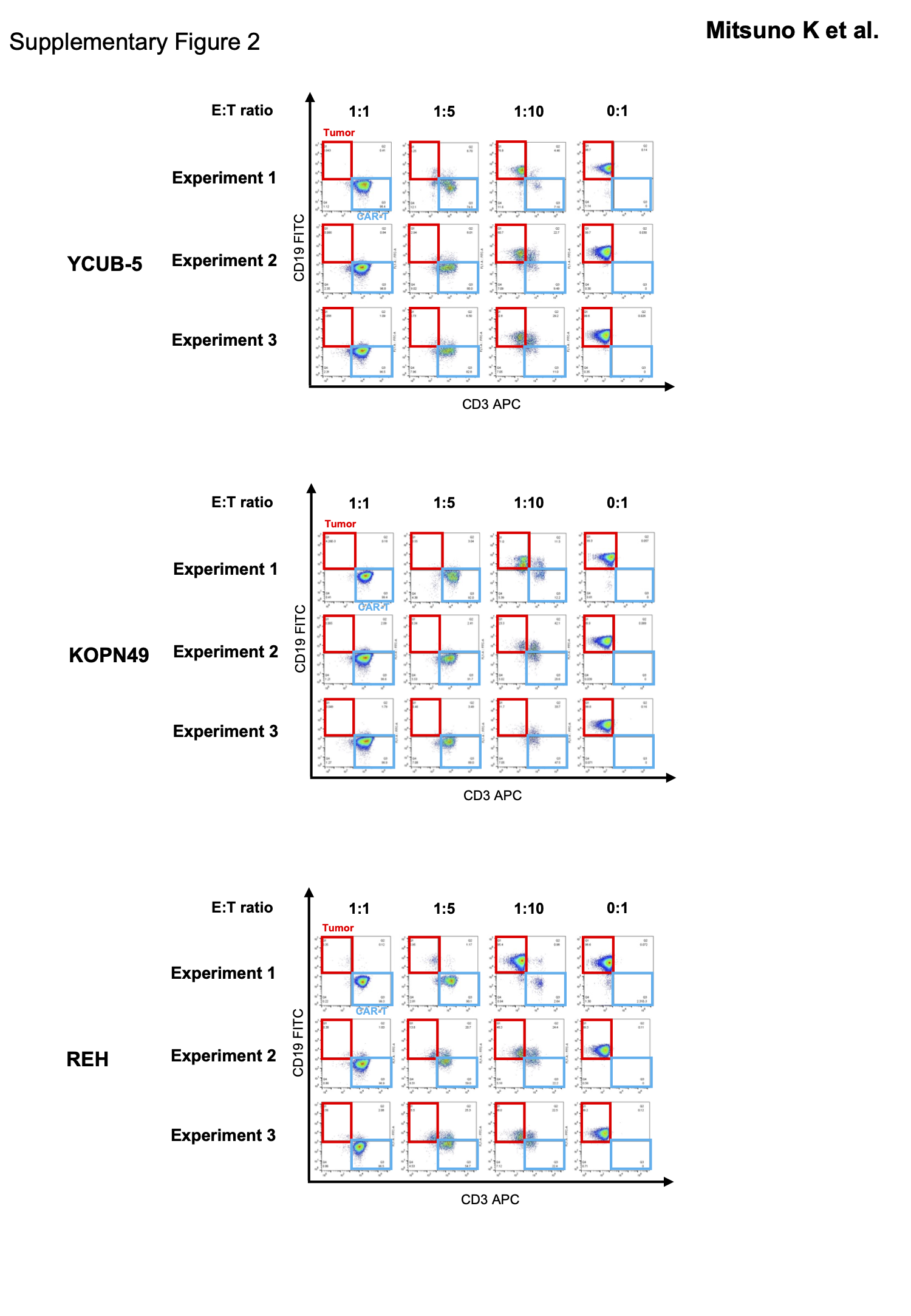

Supplement: Supplementary file 4 — Supplementary file4 [file 262_2024_3927_MOESM4_ESM.tiff]

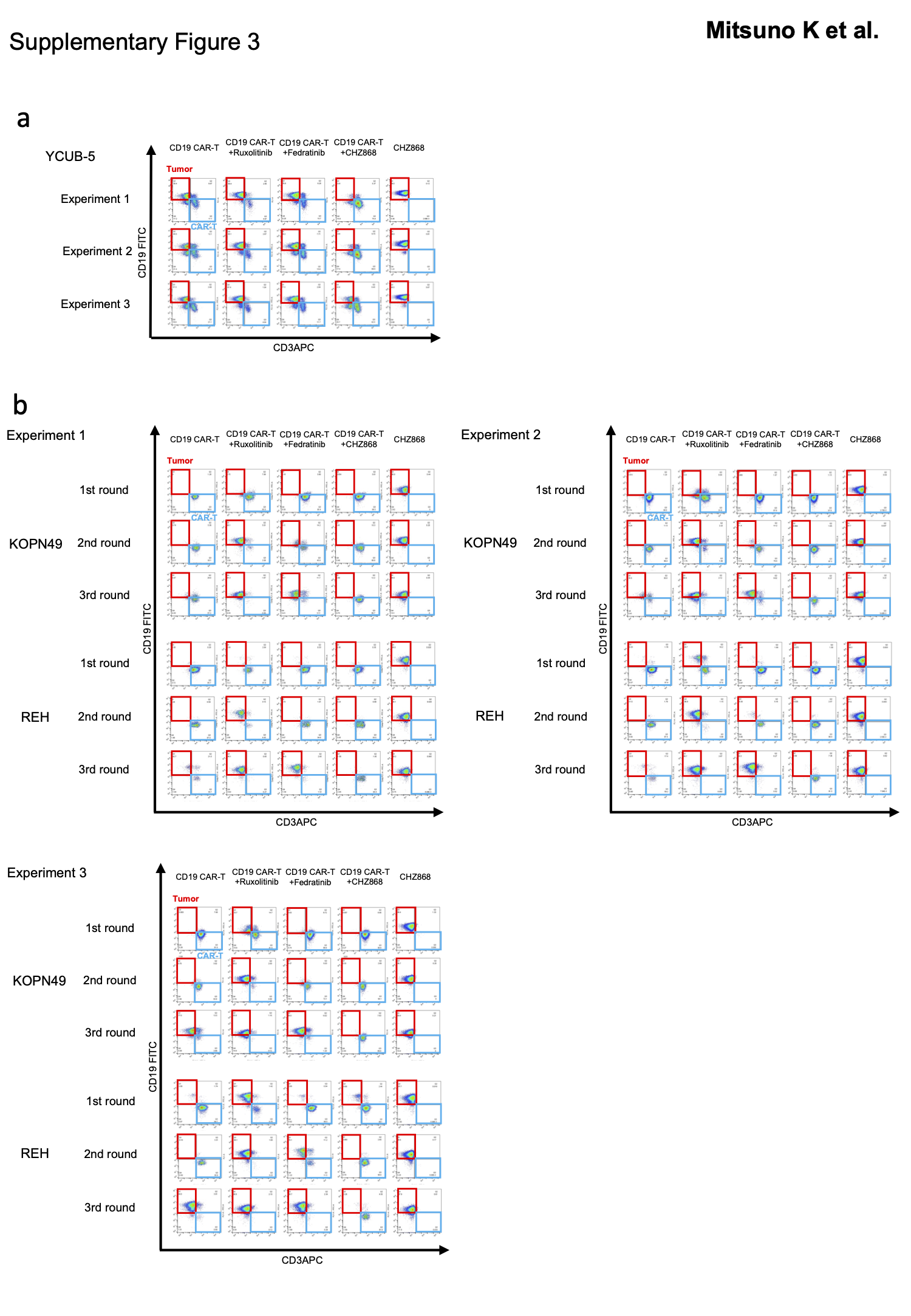

Supplement: Supplementary file 5 — Supplementary file5 [file 262_2024_3927_MOESM5_ESM.tiff]

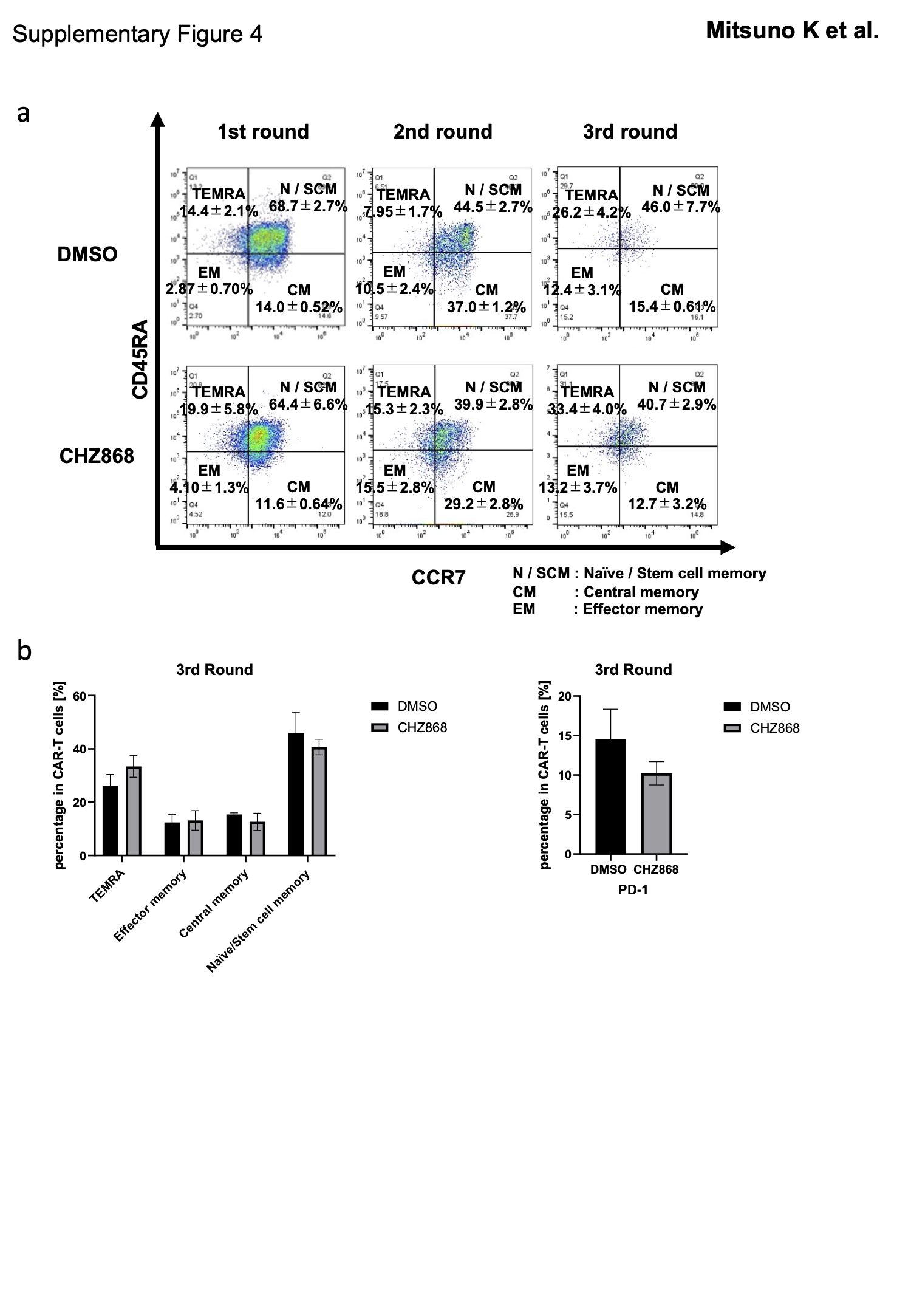

Supplement: Supplementary file 6 — Supplementary file6 [file 262_2024_3927_MOESM6_ESM.tiff]

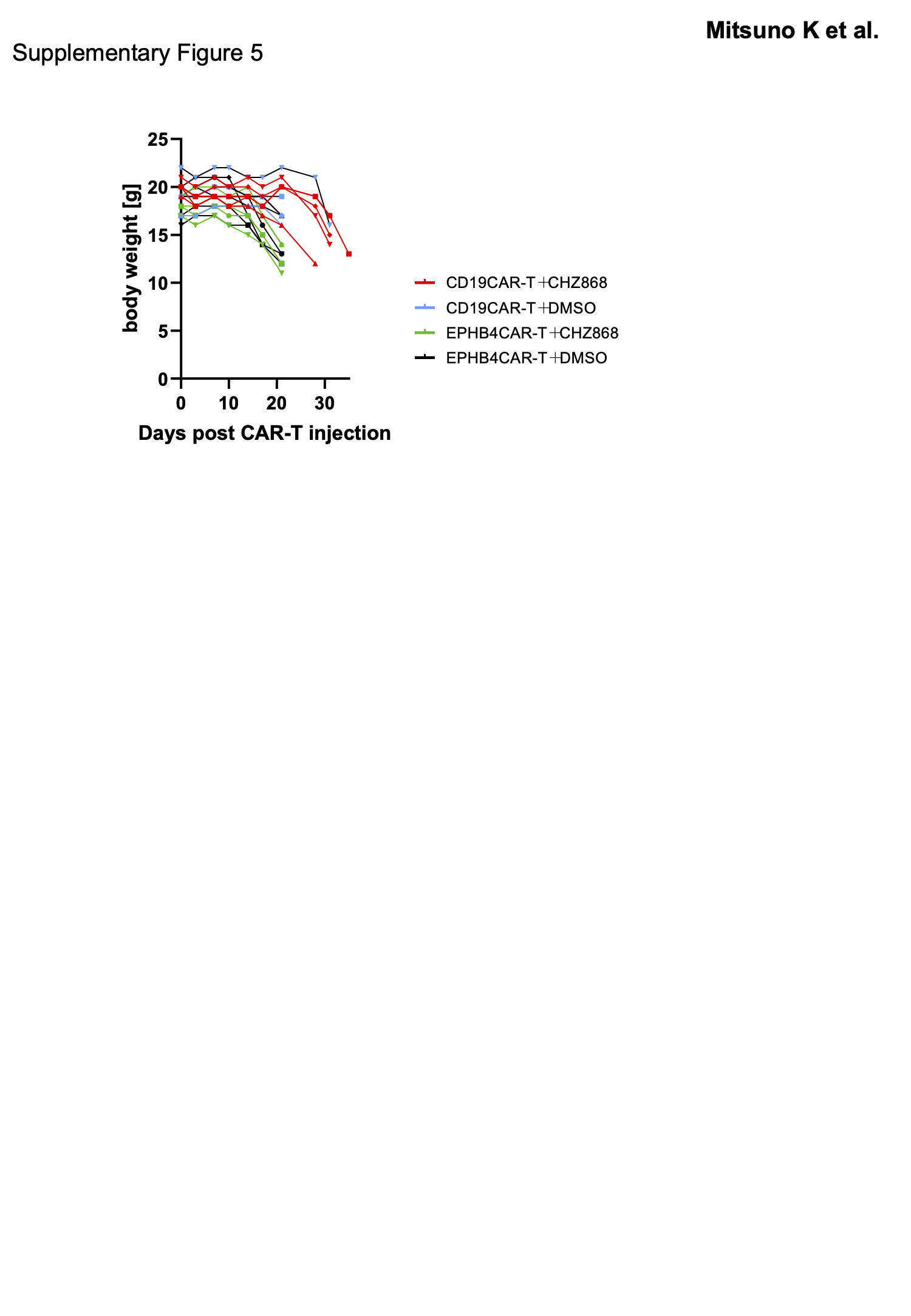

Supplement: Supplementary file 7 — Supplementary file7 [file 262_2024_3927_MOESM7_ESM.tiff]
